# *catena*-Poly[[(2,2′-bipyridine-κ^2^ *N*,*N*′)cobalt(II)]-μ-oxalato-κ^4^ *O* ^1^,*O* ^2^:*O* ^1′^,*O* ^2′^]

**DOI:** 10.1107/S1600536809037878

**Published:** 2009-09-26

**Authors:** Yan Chang, Kou-Lin Zhang, Seik Weng Ng

**Affiliations:** a College of Chemistry and Chemical Engineering, Yangzhou University, Yangzhou 225002, People’s Republic of China; b Department of Chemistry, University of Malaya, 50603 Kuala Lumpur, Malaysia

## Abstract

In the title compound, [Co(C_2_O_4_)(C_10_H_8_N_2_)]_*n*_, the oxalate group chelates two adjacent metal atoms, resulting in a zigzag chain running along the *a* axis. The Co^II^ centre exists in an all *cis*-octa­hedral coordination geometry.

## Related literature

The Mn(II), Fe(II), Ni(II), Cu(II) and Zn(II) analogs are isostructural; see: Deguenon *et al.* (1990 ▶); Fun *et al.* (1999 ▶); Lin *et al.* (2006 ▶); Luo *et al.* (2001 ▶); Yu *et al.* (2006 ▶).
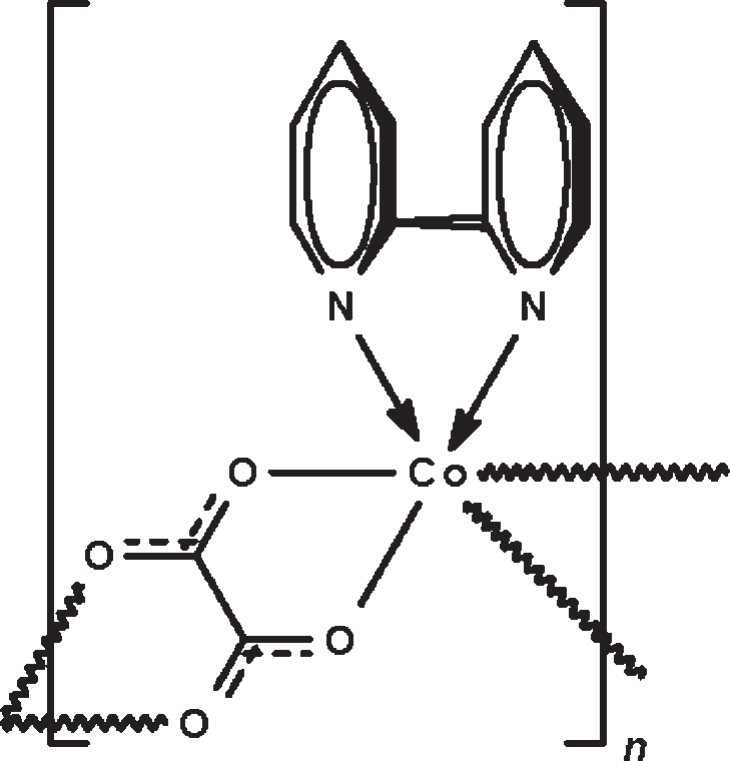


## Experimental

### 

#### Crystal data

[Co(C_2_O_4_)(C_10_H_8_N_2_)]
*M* *_r_* = 303.13
Orthorhombic, 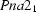

*a* = 9.1275 (8) Å
*b* = 9.2323 (8) Å
*c* = 14.1929 (12) Å
*V* = 1196.00 (18) Å^3^
*Z* = 4
Mo *K*α radiation
μ = 1.45 mm^−1^
*T* = 293 K
0.36 × 0.25 × 0.18 mm

#### Data collection

Bruker APEXII diffractometer
Absorption correction: multi-scan (*SADABS*; Sheldrick, 1996 ▶) *T*_min_ = 0.624, *T*_max_ = 0.781
9371 measured reflections
2698 independent reflections
2456 reflections with *I* > 2σ(*I*)
*R*_int_ = 0.025

#### Refinement

*R*[*F* ^2^ > 2σ(*F* ^2^)] = 0.026
*wR*(*F* ^2^) = 0.068
*S* = 1.01
2698 reflections
172 parameters
1 restraint
H-atom parameters constrained
Δρ_max_ = 0.17 e Å^−3^
Δρ_min_ = −0.23 e Å^−3^
Absolute structure: Flack (1983 ▶), 1277 Friedel pairs
Flack parameter: −0.02 (2)

### 

Data collection: *APEX2* (Bruker, 2005 ▶); cell refinement: *SAINT* (Bruker, 2005 ▶); data reduction: *SAINT*; program(s) used to solve structure: *SHELXS97* (Sheldrick, 2008 ▶); program(s) used to refine structure: *SHELXL97* (Sheldrick, 2008 ▶); molecular graphics: *X-SEED* (Barbour, 2001 ▶); software used to prepare material for publication: *publCIF* (Westrip, 2009 ▶).

## Supplementary Material

Crystal structure: contains datablocks global, I. DOI: 10.1107/S1600536809037878/bt5061sup1.cif

Structure factors: contains datablocks I. DOI: 10.1107/S1600536809037878/bt5061Isup2.hkl

Additional supplementary materials:  crystallographic information; 3D view; checkCIF report

## Figures and Tables

**Fig. 1.**
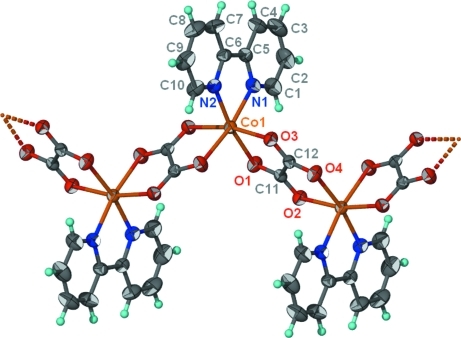
Anisotropic displacement ellipsoid plot (Barbour, 2001) of a section of the chain structure of Co(C10H8N2)(C2O4) at the 50% probability level; hydrogen atoms are drawn as spheres of arbitrary radius.

## supplementary crystallographic information

### Comment

The oxalate group chelates   two adjacent metal atoms in Co(C_10_H_8_N_2_)(C_2_O_4_)   resulting in a zigzag chain running along the *a*-axis of the orthorhombic unit cell. The cobalt atom exists in an all *cis*-octahedral coordination geometry.

### Experimental

An aqueous solution of 2*M* sodium hydroxide (0.2 ml) was added to a water/DMF (2:7 *v*/*v*) solution (9 ml) of cobalt(II) oxalate dihdyrate (0.01 g, 0.05 mmol) and 2,2'-bipyridine (0.01 g, 0.05 mmol). Pink blocks separated from the solution after several days in 30% yield. CH&N elemental analysis. Calculated for C_12_H_8_CoN_2_O_4_: C 47.55, H 2.66, N 9.24%. Found: C 47.27, H 2.94, N 9.40%.

### Refinement

Carbon-bound H-atoms were placed in calculated positions (C—H 0.93 Å) and were included in the refinement in the riding model approximation, with *U*(H) set to 1.2*U*(C).

### Crystal data

**Table:**

[Co(C_2_O_4_)(C_10_H_8_N_2_)]	*F*(000) = 612
*M**_r_* = 303.13	*D*_x_ = 1.683 Mg m^−^^3^
Orthorhombic, *P**n**a*2_1_	Mo *K*α radiation, λ = 0.71073 Å
Hall symbol: P 2c -2n	Cell parameters from 4493 reflections
*a* = 9.1275 (8) Å	θ = 2.6–26.1°
*b* = 9.2323 (8) Å	µ = 1.45 mm^−^^1^
*c* = 14.1929 (12) Å	*T* = 293 K
*V* = 1196.00 (18) Å^3^	Prism, pink
*Z* = 4	0.36 × 0.25 × 0.18 mm

### Data collection

**Table:**

Bruker APEXII diffractometer	2698 independent reflections
Radiation source: fine-focus sealed tube	2456 reflections with *I* > 2σ(*I*)
graphite	*R*_int_ = 0.025
φ and ω scans	θ_max_ = 27.5°, θ_min_ = 2.6°
Absorption correction: multi-scan (*SADABS*; Sheldrick, 1996)	*h* = −11→11
*T*_min_ = 0.624, *T*_max_ = 0.781	*k* = −11→11
9371 measured reflections	*l* = −18→18

### Refinement

**Table:**

Refinement on *F*^2^	Secondary atom site location: difference Fourier map
Least-squares matrix: full	Hydrogen site location: inferred from neighbouring sites
*R*[*F*^2^ > 2σ(*F*^2^)] = 0.026	H-atom parameters constrained
*wR*(*F*^2^) = 0.068	*w* = 1/[σ^2^(*F*_o_^2^) + (0.0436*P*)^2^] where *P* = (*F*_o_^2^ + 2*F*_c_^2^)/3
*S* = 1.01	(Δ/σ)_max_ = 0.001
2698 reflections	Δρ_max_ = 0.17 e Å^−^^3^
172 parameters	Δρ_min_ = −0.23 e Å^−^^3^
1 restraint	Absolute structure: Flack (1983), 1277 Friedel pairs
Primary atom site location: structure-invariant direct methods	Flack parameter: −0.02 (2)

### Fractional atomic coordinates and isotropic or equivalent isotropic displacement parameters (Å^2^)

**Table:**

	*x*	*y*	*z*	*U*_iso_*/*U*_eq_	
Co1	0.38104 (3)	0.58866 (2)	0.49966 (4)	0.03408 (9)	
N1	0.3016 (2)	0.4155 (2)	0.41608 (15)	0.0413 (5)	
N2	0.4481 (2)	0.40387 (19)	0.57666 (14)	0.0390 (4)	
O1	0.27899 (18)	0.75569 (19)	0.42055 (11)	0.0445 (4)	
O2	0.07533 (18)	0.88996 (17)	0.42286 (12)	0.0409 (4)	
O3	0.19137 (17)	0.61940 (16)	0.57821 (11)	0.0389 (3)	
O4	−0.00808 (15)	0.75786 (19)	0.58323 (12)	0.0449 (4)	
C1	0.2256 (3)	0.4294 (3)	0.3354 (2)	0.0551 (7)	
H1	0.1995	0.5218	0.3153	0.066*	
C2	0.1847 (3)	0.3113 (4)	0.2810 (2)	0.0674 (8)	
H2	0.1334	0.3240	0.2250	0.081*	
C3	0.2216 (4)	0.1757 (4)	0.3117 (2)	0.0710 (9)	
H3	0.1954	0.0948	0.2765	0.085*	
C4	0.2974 (3)	0.1594 (3)	0.3946 (2)	0.0574 (7)	
H4	0.3222	0.0675	0.4162	0.069*	
C5	0.3367 (3)	0.2817 (3)	0.44589 (16)	0.0385 (5)	
C6	0.4169 (3)	0.2747 (3)	0.53643 (18)	0.0390 (5)	
C7	0.4574 (4)	0.1460 (3)	0.5795 (2)	0.0583 (7)	
H7	0.4359	0.0578	0.5511	0.070*	
C8	0.5303 (4)	0.1498 (4)	0.6653 (2)	0.0661 (8)	
H8	0.5587	0.0642	0.6947	0.079*	
C9	0.5598 (3)	0.2808 (4)	0.70638 (19)	0.0635 (8)	
H9	0.6075	0.2857	0.7642	0.076*	
C10	0.5172 (3)	0.4054 (3)	0.6601 (2)	0.0531 (7)	
H110	0.5373	0.4944	0.6880	0.064*	
C11	0.1593 (2)	0.7943 (2)	0.45489 (15)	0.0329 (4)	
C12	0.1106 (2)	0.7173 (3)	0.54720 (15)	0.0329 (4)	

### Atomic displacement parameters (Å^2^)

**Table:**

	*U*^11^	*U*^22^	*U*^33^	*U*^12^	*U*^13^	*U*^23^
Co1	0.03270 (14)	0.03499 (14)	0.03454 (13)	−0.00016 (11)	0.00143 (14)	−0.00307 (16)
N1	0.0463 (11)	0.0436 (11)	0.0339 (10)	−0.0047 (8)	−0.0038 (8)	−0.0021 (8)
N2	0.0408 (11)	0.0406 (11)	0.0356 (10)	0.0037 (8)	−0.0018 (8)	−0.0021 (8)
O1	0.0411 (9)	0.0516 (10)	0.0409 (9)	0.0057 (7)	0.0145 (8)	0.0104 (8)
O2	0.0398 (8)	0.0430 (9)	0.0401 (9)	0.0032 (7)	0.0070 (7)	0.0130 (7)
O3	0.0377 (8)	0.0401 (8)	0.0388 (8)	0.0037 (7)	0.0054 (7)	0.0105 (7)
O4	0.0397 (9)	0.0536 (10)	0.0413 (9)	0.0093 (7)	0.0125 (8)	0.0179 (8)
C1	0.0618 (17)	0.0630 (17)	0.0404 (13)	−0.0075 (13)	−0.0131 (12)	−0.0006 (12)
C2	0.0740 (19)	0.085 (2)	0.0435 (15)	−0.0175 (17)	−0.0170 (14)	−0.0103 (14)
C3	0.082 (2)	0.069 (2)	0.0628 (18)	−0.0255 (17)	−0.0066 (16)	−0.0259 (16)
C4	0.0692 (18)	0.0465 (15)	0.0565 (16)	−0.0145 (13)	−0.0005 (14)	−0.0107 (12)
C5	0.0407 (11)	0.0403 (12)	0.0346 (12)	−0.0044 (11)	0.0034 (10)	−0.0051 (10)
C6	0.0429 (12)	0.0341 (11)	0.0402 (12)	0.0035 (10)	0.0077 (10)	−0.0023 (9)
C7	0.0777 (19)	0.0428 (14)	0.0544 (15)	0.0149 (14)	−0.0004 (15)	−0.0044 (13)
C8	0.086 (2)	0.0576 (17)	0.0546 (16)	0.0271 (16)	0.0024 (16)	0.0105 (15)
C9	0.0727 (18)	0.077 (2)	0.0406 (15)	0.0236 (16)	−0.0064 (13)	0.0052 (14)
C10	0.0622 (17)	0.0555 (16)	0.0416 (13)	0.0124 (12)	−0.0082 (13)	−0.0062 (11)
C11	0.0342 (10)	0.0332 (11)	0.0312 (10)	−0.0055 (10)	0.0024 (9)	0.0017 (9)
C12	0.0318 (11)	0.0349 (11)	0.0321 (11)	−0.0025 (9)	0.0030 (8)	0.0025 (9)

### Geometric parameters (Å, °)

**Table:**

Co1—O3	2.0786 (16)	C1—H1	0.9300
Co1—O2^i^	2.0909 (16)	C2—C3	1.367 (5)
Co1—O4^i^	2.1068 (16)	C2—H2	0.9300
Co1—N1	2.119 (2)	C3—C4	1.374 (5)
Co1—N2	2.1165 (19)	C3—H3	0.9300
Co1—O1	2.1228 (17)	C4—C5	1.391 (4)
N1—C1	1.344 (3)	C4—H4	0.9300
N1—C5	1.344 (3)	C5—C6	1.480 (3)
N2—C10	1.342 (3)	C6—C7	1.386 (4)
N2—C6	1.352 (3)	C7—C8	1.388 (4)
O1—C11	1.248 (3)	C7—H7	0.9300
O2—C11	1.255 (3)	C8—C9	1.369 (5)
O2—Co1^ii^	2.0909 (16)	C8—H8	0.9300
O3—C12	1.247 (3)	C9—C10	1.381 (4)
O4—C12	1.255 (2)	C9—H9	0.9300
O4—Co1^ii^	2.1068 (16)	C10—H110	0.9300
C1—C2	1.388 (4)	C11—C12	1.555 (2)
			
O3—Co1—O2^i^	166.68 (6)	C2—C3—C4	119.8 (3)
O3—Co1—O4^i^	90.36 (6)	C2—C3—H3	120.1
O2^i^—Co1—O4^i^	79.78 (6)	C4—C3—H3	120.1
O3—Co1—N1	96.78 (8)	C3—C4—C5	119.3 (3)
O2^i^—Co1—N1	94.00 (7)	C3—C4—H4	120.4
O4^i^—Co1—N1	170.80 (7)	C5—C4—H4	120.4
O3—Co1—N2	94.24 (7)	N1—C5—C4	121.3 (2)
O2^i^—Co1—N2	95.74 (7)	N1—C5—C6	115.6 (2)
O4^i^—Co1—N2	96.46 (8)	C4—C5—C6	123.1 (3)
N1—Co1—N2	77.29 (7)	N2—C6—C7	120.9 (3)
O3—Co1—O1	79.57 (6)	N2—C6—C5	115.6 (2)
O2^i^—Co1—O1	91.60 (7)	C7—C6—C5	123.5 (3)
O4^i^—Co1—O1	91.15 (7)	C8—C7—C6	119.6 (3)
N1—Co1—O1	95.83 (8)	C8—C7—H7	120.2
N2—Co1—O1	170.25 (7)	C6—C7—H7	120.2
C1—N1—C5	118.6 (2)	C9—C8—C7	119.3 (3)
C1—N1—Co1	125.54 (18)	C9—C8—H8	120.3
C5—N1—Co1	115.80 (17)	C7—C8—H8	120.3
C10—N2—C6	118.8 (2)	C8—C9—C10	118.6 (3)
C10—N2—Co1	125.67 (16)	C8—C9—H9	120.7
C6—N2—Co1	115.58 (17)	C10—C9—H9	120.7
C11—O1—Co1	112.63 (14)	N2—C10—C9	122.9 (3)
C11—O2—Co1^ii^	113.30 (14)	N2—C10—H110	118.5
C12—O3—Co1	113.72 (14)	C9—C10—H110	118.5
C12—O4—Co1^ii^	112.70 (15)	O1—C11—O2	126.4 (2)
N1—C1—C2	122.5 (3)	O1—C11—C12	116.67 (18)
N1—C1—H1	118.8	O2—C11—C12	116.88 (17)
C2—C1—H1	118.8	O3—C12—O4	125.7 (2)
C3—C2—C1	118.5 (3)	O3—C12—C11	117.35 (17)
C3—C2—H2	120.8	O4—C12—C11	116.98 (18)
C1—C2—H2	120.8		
			
O3—Co1—N1—C1	86.2 (2)	C1—N1—C5—C6	−178.5 (2)
O2^i^—Co1—N1—C1	−86.0 (2)	Co1—N1—C5—C6	4.1 (3)
N2—Co1—N1—C1	179.0 (2)	C3—C4—C5—N1	0.2 (4)
O1—Co1—N1—C1	6.0 (2)	C3—C4—C5—C6	179.3 (3)
O3—Co1—N1—C5	−96.68 (18)	C10—N2—C6—C7	−1.1 (4)
O2^i^—Co1—N1—C5	91.16 (18)	Co1—N2—C6—C7	179.0 (2)
N2—Co1—N1—C5	−3.84 (18)	C10—N2—C6—C5	178.2 (2)
O1—Co1—N1—C5	−176.83 (18)	Co1—N2—C6—C5	−1.7 (3)
O3—Co1—N2—C10	−81.0 (2)	N1—C5—C6—N2	−1.6 (3)
O2^i^—Co1—N2—C10	90.2 (2)	C4—C5—C6—N2	179.2 (3)
O4^i^—Co1—N2—C10	9.8 (2)	N1—C5—C6—C7	177.7 (3)
N1—Co1—N2—C10	−177.0 (2)	C4—C5—C6—C7	−1.5 (4)
O3—Co1—N2—C6	98.92 (19)	N2—C6—C7—C8	0.4 (5)
O2^i^—Co1—N2—C6	−89.91 (18)	C5—C6—C7—C8	−178.9 (2)
O4^i^—Co1—N2—C6	−170.23 (18)	C6—C7—C8—C9	0.5 (5)
N1—Co1—N2—C6	2.91 (19)	C7—C8—C9—C10	−0.7 (5)
O3—Co1—O1—C11	0.09 (16)	C6—N2—C10—C9	0.9 (4)
O2^i^—Co1—O1—C11	−169.87 (16)	Co1—N2—C10—C9	−179.2 (2)
O4^i^—Co1—O1—C11	−90.06 (16)	C8—C9—C10—N2	0.0 (4)
N1—Co1—O1—C11	95.94 (17)	Co1—O1—C11—O2	−179.71 (19)
O2^i^—Co1—O3—C12	47.6 (4)	Co1—O1—C11—C12	1.1 (2)
O4^i^—Co1—O3—C12	89.55 (17)	Co1^ii^—O2—C11—O1	177.06 (19)
N1—Co1—O3—C12	−96.27 (17)	Co1^ii^—O2—C11—C12	−3.8 (2)
N2—Co1—O3—C12	−173.95 (17)	Co1—O3—C12—O4	−178.58 (19)
O1—Co1—O3—C12	−1.55 (16)	Co1—O3—C12—C11	2.6 (2)
C5—N1—C1—C2	−1.3 (4)	Co1^ii^—O4—C12—O3	−174.00 (19)
Co1—N1—C1—C2	175.8 (2)	Co1^ii^—O4—C12—C11	4.9 (2)
N1—C1—C2—C3	0.9 (5)	O1—C11—C12—O3	−2.6 (3)
C1—C2—C3—C4	0.0 (5)	O2—C11—C12—O3	178.2 (2)
C2—C3—C4—C5	−0.5 (5)	O1—C11—C12—O4	178.4 (2)
C1—N1—C5—C4	0.7 (4)	O2—C11—C12—O4	−0.8 (3)
Co1—N1—C5—C4	−176.7 (2)		

Symmetry codes: (i) *x*+1/2, −*y*+3/2, *z*; (ii) *x*−1/2, −*y*+3/2, *z*.
